# Glucokinase Deficit Prevalence in Women With Diabetes in Pregnancy: A Matter of Screening Selection

**DOI:** 10.3389/fendo.2020.00268

**Published:** 2020-05-20

**Authors:** Olimpia Bitterman, Chiara Giuliani, Camilla Festa, Angela Napoli

**Affiliations:** ^1^Department of Clinical and Molecular Medicine, Sant'Andrea Hospital, Sapienza University of Rome, Rome, Italy; ^2^Department of Experimental Medicine, Sant'Andrea Hospital, Sapienza University of Rome, Rome, Italy

**Keywords:** monogenic diabetes mellitus, glucokinase (GCK) gene mutation, gestational diabetes, MODY, hyperglycemia

## Abstract

**Introduction:** The prevalence among pregnant women with diabetes of monogenic diabetes due to glucokinase deficit (GCK-MODY) varies from 0 to 80% in different studies, based on the chosen selection criteria for genetic test. New pregnancy-specific Screening Criteria (NSC), validated on an Anglo-Celtic pregnant cohort, have been proposed and include pre-pregnancy BMI <25 kg/m^2^ and fasting glycemia >99 mg/dl. Our aim was to estimate the prevalence of GCK-MODY and to evaluate the diagnostic performance of NSC in our population of women with diabetes in pregnancy.

**Patients and Methods:** We retrospectively selected from our database of 468 diabetic pregnant patients in Sant'Andrea Hospital, in Rome, from 2010 to 2018, all the women who received a genetic test for GCK deficit because of specific clinical features. We estimated the prevalence of GCK-MODY among tested women and the minimum prevalence in our entire population with non-autoimmune diabetes. We evaluated diagnostic performance of NSC on the tested cohort and estimated the eligibility to genetic test based on NSC in the entire population.

**Results:** A total of 409 patients had diabetes in pregnancy, excluding those with autoimmune diabetes; 21 patients have been tested for GCK-MODY, 8 have been positive and 13 have been negative (2 of them had HNF1-alfa mutations and 1 had HNF4-alfa mutation). We found no significant differences in clinical features between positive and negative groups except for fasting glycemia, which was higher in the positive group. The minimum prevalence of monogenic diabetes in our population was 2.4%. The minimum prevalence of GCK-MODY was 1.95%. In the tested cohort, the prevalence of GCK-MODY was 38%. In this group, NSC sensitivity is 87% and specificity is 30%, positive predictive value is 43%, and negative predictive value is 80%. Applying NSC on the entire population of women with non-autoimmune diabetes in pregnancy, 41 patients (10%) would be eligible for genetic test; considering a fasting glycemia >92 mg/dl, 85 patients (20.7%) would be eligible.

**Discussion:** In our population, NSC have good sensitivity but low specificity, probably because there are many GDM with GCK-MODY like features. It is mandatory to define selective criteria with a good diagnostic performance on Italian population, to avoid unnecessary genetic tests.

## Introduction

Pregnancy is often the first chance for a young woman to evaluate her glycemia, so monogenic diabetes due to glucokinase deficit (GCK-MODY) can be first detected during pregnancy, although hyperglycemia is present from birth (1). Unfortunately, it is often misdiagnosed with gestational diabetes (GDM) or type 2 diabetes, because of its rarity and because of the logistic problems to obtain the genetic test, which is expensive and not available everywhere.

GCK-MODY prevalence among women with GDM varies from 0 to 6%, based on the clinical criteria chosen to test the patients (2–11) (see Table 1). A study on Caucasian English women with diabetes in pregnancy found a prevalence of 80%, using stricter criteria (12).

**Table 1:** GCK-MODY prevalence in pregnant women with hyperglycemia.

**Authors**	**Selected cohort to test**	**Prevalence**
Stoffel et al. (2)	40 American women (different ethnicities) with GDM and a 1st-degree relative with diabetes	5%
Zouali et al. (8)	17 French women with GDM and familiarity for type 2 diabetes	6%
Saker et al. (3)	50 English women with GDM and persistent hyperglycemia	6%
Ellard et al. (12)	15 English Caucasian women with GDM and specific criteria (persistent fasting hyperglycemia >99 mg/dl, 2-h glycemic increase in OGTT <83 mg/dl, insulin therapy in pregnancy but not after pregnancy and fasting hyperglycemia or diabetes in at least one 1st-degree relative)	80%
Kousta et al. (5)	17 women (different ethnicities) with specific criteria (persistent fasting hyperglycemia >99 mg/dl, 2-h glycemic increase in postpartum OGTT <63 mg/dl)	12%
Weng et al. (4)	66 Swedish women with con GDM and familiarity for diabetes	2% (3% other types of monogenic diabetes)
Zurawek et al. (6)	119 Polish women with GDM and specific criteria (age <35 years, pregestational BMI <25 kg/m^2^, 2-h glycemic increase in OGTT <83 mg/dl, insulin therapy in pregnancy but not after pregnancy and fasting hyperglycemia or diabetes in at least one 1st-degree relative)	9%
Chakera et al. (7)	356 pregnant Anglo-Celtic women who performed OGTT	1%
Sewell et al. (9)	72 women with GDM	0%
Rudland et al. (10)	31 Australian women (different ethnicities) selected by Chakera pregnancy-specific criteria (7)	12.9%
Gjesing et al. (11)	354 Danish women with GDM treated with diet	1.9%

GCK-MODY diagnosis in pregnancy could lead to a different treatment and follow up of the patient (1, 13, 14) and could avoid a “wrong” diagnosis of type 1 diabetes in the offspring (who will inherit diabetes in 50% of cases). The gold standard for GCK-MODY diagnosis is the genetic test, but because of its high cost, it is important to select high-risk patients. In 2008, Ellard et al. (15) proposed the following selection criteria, which had a sensitivity of 98% on their population: fasting hyperglycemia ≥99 mg/dl, persistent (at least three times) and stable in time; HbA1c slightly higher than normal but usually not over 7.5%; small glycemic increase in OGTT (<83 mg/dl); parents with not complicated type 2 diabetes or apparently not diabetic (15).

However, selection criteria validated on non-pregnant patients could not be appropriate for pregnant women, because of the gestational physiological changes in glucose metabolism. For this reason, Chakera et al. (7) proposed New pregnancy-specific Screening Criteria (NSC) for GCK-MODY suspect (7). They first evaluated GCK-MODY prevalence in a cohort of women from the “Atlantic DIP” study (16), which was 1%; then, based on the clinical features of these patients, they identified pregestational BMI and fasting glycemia as useful selection criteria for GCK-MODY test. In particular, the combination of pregestational BMI <25 kg/m^2^ and fasting glycemia >99 mg/dl had the best diagnostic performance, with a sensitivity of 68% and a specificity of 99%, needing 2.7 genetic test to find 1 case of GCK-MODY. Choosing different BMI cutoff, sensitivity and specificity vary, up to 100% of specificity with BMI <21 kg/m^2^. Since they did not find any case of GCK-MODY in women with normal fasting glucose and only 1 case in women with fasting glucose between 92 and 99 mg/dl, they only considered fasting glycemia >99 mg/dl in their analysis.

Later, Rudland et al. (10) evaluated NSC and pregestational HbA1C as selection criteria in their population of Australian women with GDM, comparing them with Ellard standard criteria. They found that NSC selected 14.6% of patients vs. 6.1% with standard criteria. Anyway, NSC selected all the Anglo-Celtic women but not all the Indian women actually affected by GCK-MODY. Concerning HbA1C, it was not useful at all to distinguish affected patients from non-affected ones.

The aims of our study were (1) to estimate the minimum prevalence of GCK-MODY in our population of diabetic pregnant women, excluding those with autoimmune diabetes; (2) to evaluate the diagnostic performance of NSC in a cohort of women tested for GCK-MODY; and (3) to explore the eligibility to genetic test based on NSC in our entire population of pregnant women with diabetes in pregnancy.

## Patients and Methods

We retrospectively analyzed data from 468 diabetic pregnant patients who referred to “Diabetes and Pregnancy” outpatients' office in Sant'Andrea Hospital, in Rome, from 2010 to 2018. We selected from our database all the women who received a genetic test for GCK-MODY. We selected the patients to test case by case over time, based on the clinical suspicion and the availability of the genetic test in that moment, but essential features to be selected were negative GAD antibodies and fasting hyperglycemia at the first visit in pregnancy ≥92 mg/dl.

We estimated the prevalence of GCK-MODY in this cohort of tested women and the minimum prevalence in our entire population of patients with diabetes in pregnancy. Furthermore, we applied NSC (pregestational BMI <25 kg/m^2^ and gestational fasting glycemia >99 mg/dl) on the tested cohort to evaluate their diagnostic performance; we also estimated the eligibility to genetic test based on NSC in our entire population of diabetic pregnant women with negative GAD autoantibodies.

### Statistical Analysis

Quantitative variables were expressed as median and interquartile range (25%–75%) or mean and standard deviation, when appropriate; qualitative variables were expressed as absolute number or percentages. We used Mann Whitney test or Student *t*-test for comparison between groups with quantitative variables, according to data distribution, while χ^2^ test was used for frequency comparisons. Only *p*-values < 0.05 were considered significant. Data were processed using the IBM program “SPSS 20.”

## Results

From 2010 to 2018, we followed 409 patients with diabetes in pregnancy with negative GAD antibodies, including 356 women with diagnosis of GDM [diagnosed with IADPS criteria (17)], 7 pregestational impaired fasting glucose (IFG) and 36 pregestational type 2 diabetes. Genetic test for GCK mutations has been performed on 21 patients, 8 have been positive (3 GDM, 1 IGF, and 4 type 2 diabetes) and 13 have been negative (10 GDM, 2 IFG, and 1 type 2 diabetes). Subsequently, because of strong clinical suspect, 3 women from the negative group have been tested for other types of monogenic diabetes, resulting in 2 HNF1-alfa mutations and 1 HNF4-alfa mutation (this last case was not clearly related to hyperglycemia in the literature).

### Clinical Features

The clinical features of the entire population are shown in Table 2. Only 49.5% of the women was overweight (26.8% with BMI 25–30 kg/m^2^ and 22.7% with BMI >30 kg/m^2^).

**Table 2:** Clinical features in terms of pregestational BMI and fasting glycemia in pregnancy of the entire population of pregnant diabetes women with negative GAD antibodies.

	**BMI <25 (*n* = 205) 50.1%**	**BMI = 25–30 (*n* = 110) 26.8%**
Glycemia > 92 mg/dl, *n* (%)	85 (41.4)	61 (55.4)
Glycemia >99 mg/dl, *n* (%)	41 (20)	29 (26.3)

Among the tested women, we compared clinical features between positive and negative for GCK-MODY and found no significant differences, except for fasting glycemia median at the first visit in pregnancy, which is significantly higher in the positive group (see Table 3). Fasting glycemia ranged from 98 to 130 mg/dl in the positive group and from 92 to 107 mg/dl in the negative group. All the women had a pregestational BMI <25 kg/m^2^ except for one woman in the negative group who had BMI = 25.1 kg/m^2^.

**Table 3:** Clinical features of the women tested for GCK-MODY, based on the result of the genetic test.

	**Positive (*n* = 8)**	**Negative (*n* = 13)**	***P*-value**
Age (years)	31.9 ± 5.6	34 ± 3.6	NS
Pregestational BMI (kg/m^2^)	23.2 (21.6–24.1)	23.6 (21.5–24.3)	NS
1st-degree relative with hyperglycemia (%)	100%	80%	NS
Fasting glycemia at the first visit in pregnancy (mg/dl)	111 (103.25–121)	96 (93–100)	<0.01
Fasting glycemia > 99 mg/dl at the first visit in pregnancy, *n* (%)	7 (87.5)	9 (69.2)	NS

### Prevalence of Monogenic Diabetes and Glucokinase Deficit

The minimum prevalence of monogenic diabetes in our population was 2.4%, considering the two women with HNF1-alfa mutation.

In a cohort of 409 patients with diabetes in pregnancy and negative GAD antibodies, the minimum prevalence of GCK-MODY was 1.95%. In the cohort of 21 tested women, arbitrarily selected by clinical features, the prevalence of GCK-MODY was 38%.

### NSC Diagnostic Performance

Applying NSC to our tested cases of GCK-MODY, they identify only 7 of 8 positive patients and 9 of 13 of negative patients, because 1 of the positive and 4 of the negative women had a fasting glycemia at the first visit in pregnancy <99 mg/dl (the positive had 98 mg/dl and the negatives had 92 to 96 mg/dl).

Consequently, in our tested cohort of patients, NSC sensitivity is 87% and specificity is 30%, with a positive predictive value of 43% and a negative predictive value of 80%. Applying NSC on the entire population of women with diabetes in pregnancy and negative GAD antibodies, 41 patients of 409 (10%) would be eligible for genetic test. Applying the same criteria but choosing a BMI <30 kg/m^2^ (instead of 25 kg/m^2^), 70 patients (17.1%) would be eligible.

When considering a fasting glycemia >92 mg/dl (instead of 99 mg/dl), which is the pregnancy-specific fasting cutoff for diabetes diagnosis (17), 85 patients (20.7%) with BMI <25 kg/m^2^ and 146 (35.7%) with BMI <30 kg/m^2^ would be eligible for the test.

## Discussion

In our population with diabetes in pregnancy and negative GAD antibodies, the minimum prevalence of GCK-MODY is 1.95%. It is important to notice that we randomly tested only women with specific and arbitrary criteria; in this group, the prevalence is 38%. In other studies, the prevalence of GCK-MODY in specific group with different selection criteria varies from 0 to 80%. We did not use the criteria of diabetes familiarity, as in many of these studies, because patients are often unaware of this information and/or because the slightly higher glycemia in relatives could remain unnoticed.

The limitation of our study is that we were not able to test all the women with our chosen selected criteria, because of the high price and difficult availability of the genetic test, so the prevalence could be underestimated. Moreover, the choice of the patients to test over the years was arbitrary and not structured, with the bias of the typical definition of monogenic diabetes (normal BMI, familiarity), so we could have missed many diagnoses with atypical features (especially in overweight patients).

Applying Chakera's pregnancy-specific criteria to our cohort of tested women, sensitivity is higher but specificity is lower than in the Atlantic DIP cohort, probably meaning that in our population, there are many GDM patients with GCK-MODY-like features. In fact, our patients without GCK-MODY do not differ from affected patients for BMI or diabetes familiarity.

NSC would select a high percentage of our patients (10%), higher when considering a lower cutoff of fasting hyperglycemia (92 mg/dl, the specific pregnancy cutoff for diabetes diagnosis), because of the high number of normal weight women with fasting hyperglycemia. It has to be noticed that, in our entire population, half of the patients are not overweight or obese, as typically expected in women with GDM or type 2 diabetes. It could mean that in these women, hyperglycemia pathogenesis is different from GDM and type 2 diabetes. Another variable could be that the Anglo-Celtic population of the Atlantic Dip study, on which Chakera evaluated his selection criteria, is different from our Italian population in terms of weight excess prevalence (18, 19), so BMI criteria may not apply in the same way to both populations. Consequently, typical selection criteria for monogenic diabetes screening, such as normal BMI and strong diabetes familiarity, could not be applied to these kinds of patients.

## Conclusions

GCK-MODY represents a challenging diagnosis for clinicians, but it is important to guide clinical choices in terms of therapy and follow-up not only for pregnant women, but also for their offspring. Since genetic test for GCK-MODY is very expensive and not available everywhere, leading to logistic problems as well, it is mandatory to define selective criteria with a good diagnostic performance on an Italian population, to avoid an excessive increase in health expenditure that is not cost-effective.

## Data Availability Statement

The raw data supporting the conclusions of this article will be made available by the authors, without undue reservation, to any qualified researcher.

## Ethics Statement

Ethical review and approval was not required for the study on human participants in accordance with the local legislation and institutional requirements. Written informed consent for participation was not required for this study in accordance with the national legislation and the institutional requirements.

## Author Contributions

OB and AN: conceptualization, software, validation, writing—review and editing, and visualization. AN: methodology, supervision, and project administration. CF: formal analysis. OB, CG, and CF: investigation and data curation. OB: resources and writing—original draft preparation.

## Conflict of Interest

The authors declare that the research was conducted in the absence of any commercial or financial relationships that could be construed as a potential conflict of interest. The reviewer DI declared a past co-authorship with the authors OB and AN.
